# Observing Protein One-Dimensional Sliding: Methodology and Biological Significance

**DOI:** 10.3390/biom11111618

**Published:** 2021-11-02

**Authors:** Xiao-Wen Yang, Jiaquan Liu

**Affiliations:** State Key Laboratory of Molecular Biology, CAS Center for Excellence in Molecular Cell Science, Shanghai Institute of Biochemistry and Cell Biology, University of Chinese Academy of Sciences, Chinese Academy of Sciences, Shanghai 200031, China; yangxiaowen2019@sibcb.ac.cn

**Keywords:** DNA-binding protein, one-dimensional sliding, single molecule

## Abstract

One-dimensional (1D) sliding of DNA-binding proteins has been observed by numerous kinetic studies. It appears that many of these sliding events play important roles in a wide range of biological processes. However, one challenge is to determine the physiological relevance of these motions in the context of the protein’s biological function. Here, we discuss methods of measuring protein 1D sliding by highlighting the single-molecule approaches that are capable of visualizing particle movement in real time. We also present recent findings that show how protein sliding contributes to function.

## 1. Introduction

The interactions between proteins and DNA are involved in most cellular functions of DNA, including DNA replication, DNA repair and recombination, and gene transcription and regulation. Many if not all protein–DNA interactions are essential for those processes. When the physical properties of genomic DNA have been well described by a worm-like chain model [1,2], a number of biophysical studies have provided evidence of protein performing one-dimensional (1D) sliding along the DNA [3,4,5,6]. These highly dynamic protein–DNA interactions have served as models for understanding the macromolecular interactions in biology.

The concept of protein 1D sliding has been recognized for several decades. DNA/RNA polymerases and helicases that operate one nucleotide after another are early examples that obviously must move in 1D along a DNA strand [7,8]. Further investigations have found that many sequence-specific DNA-binding proteins, including a number of restriction endonucleases and transcription factors, also slide along the double-stranded DNA (dsDNA) to search for target sites [9,10,11]. While most studies have inspected protein 1D sliding using relatively simple systems, many complicated biological questions involve protein complex assembly and multiple reaction steps that could not be assessed with these systems. In this review, we have summarized recent studies of protein 1D sliding. Several modes of the sliding and the methods for measuring protein movement have been introduced and discussed here. Of particular interest, we highlight the observations that have ultimately revealed the biological functions of protein 1D sliding.

## 2. Modes of Protein 1D Sliding

### 2.1. Translocation

**Figure 1:**
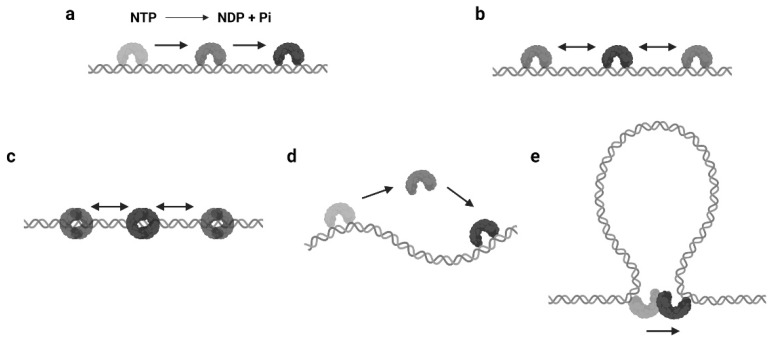
Modes of protein 1D sliding. (**a**) Translocation: protein moves along DNA in a unidirectional way by NTP hydrolysis (NDP: nucleoside diphosphate, Pi: inorganic phosphate). (**b**) Facilitated diffusion: protein passively slides on DNA to search targets after nonspecific binding. (**c**) Sliding as a clamp: ring-like protein passively slides on DNA with intermittent DNA contacts. (**d**) Hopping or jumping: protein diffuses on DNA with a series of disassociation and rebinding events. (**e**) Intersegment transfer: protein moves from one binding site to another via the formation of a transient DNA loop. The double helix structures represent dsDNA and the arch or ring-like structures represent DNA-binding proteins. The black arrows indicate the directions of protein movements on DNA.

The mechanisms of protein 1D sliding can be categorized into several modes. Probably the most familiar one for biologists is translocation. The majority of DNA/RNA polymerases and helicases hydrolyze nucleoside triphosphate (NTP) and have been suggested to use the released energy for moving along DNA in a unidirectional manner [12,13,14,15]. This unidirectional movement is defined as translocation (Figure 1a). Moreover, there are other proteins that also translocate along the DNA by NTP hydrolysis but do not couple the directional motions to the nucleotide incorporation or strand separation activity [16]. Those enzymes are referred as translocases. As it can be seen, the translocation is an active motion, and the proteins that perform translocation may be considered as molecular motors on DNA [17,18]. While the protein translocation activity certainly requires NTP hydrolysis, the reverse is not necessarily true—a protein that performs 1D sliding and contains NTPase activity may not translocate along the DNA (see Section 2.3).

### 2.2. Facilitated Diffusion

In biophysical studies, three-dimensional (3D) diffusion and random collision are usually used to describe and predict the searching dynamics of two biomolecules that interact with each other. Interestingly, it was reported that the lacI repressor could bind its target sequence about two orders of magnitude faster than the predicted [3,19]. Further analysis found that lacI was capable of nonspecifically binding to DNA by electrostatic interactions and then passively sliding on DNA to locate its specific binding site [20,21]. This progression is termed as facilitated diffusion, where the search process has been “facilitated” by protein 1D sliding (Figure 1b). Since then, many other enzymes have been found that utilize this mechanism for a target search, including restriction endonucleases and other transcription factors.

### 2.3. Sliding as a Clamp

The continuous protein–DNA contacts without dissociation are usually necessary to support facilitated diffusion. However, there are proteins that obviously slide with intermittent DNA contacts. Members of this group include proteins that can form special conformations on DNA, such as a clamp encircling the DNA (Figure 1c). A classic example is the DNA replication factor (β-clamp in prokaryotes, also known as proliferating cell nuclear antigen, or PCNA, in eukaryotes), which is a ring-shaped clamp and requires a clamp loader to load it onto the DNA [22,23]. Coincidentally, the DNA mismatch repair (MMR) proteins, MutS and MutL homologs, also form ATP-dependent ring-like clamps during MMR. These sliding clamps do not always interact with the DNA backbone but can maintain a relatively long lifetime (several minutes) during sliding [24,25,26,27]. It is now well established that the MutS and MutL sliding clamps are actually molecular switches that undergo Brownian motion along the DNA, while their ring-like structures prevent the proteins from disassociating from the DNA [24,27].

### 2.4. Hopping or Jumping

Another type of protein sliding with intermittent DNA contacts is hopping/jumping, where the protein diffuses with a series of disassociation and rebinding events [3] (Figure 1d). Compared with the facilitated diffusion, one could imagine the protein may slide faster and is able to bypass obstacles during hopping/jumping. As a result, proteins combining facilitated diffusion and hopping/jumping seem to be more efficient when searching for target sites [28]. However, distinct from the sliding clamps that are topologically constrained to the DNA, it is now still unclear how the hopping/jumping occurs.

### 2.5. Intersegmental Transfer

In some scenarios, large DNA molecules can form transient DNA loops. A few proteins are capable of moving between two sites on DNA via these intermediate “loops”, which is referred to as intersegmental transfer (Figure 1e). Intersegmental transfer allows the protein to move from one site of DNA to another without dissociation, thus facilitating the target search [3]. However, intersegmental transfer is more likely to occur when the protein contains two or more DNA binding surfaces, since the two binding sites need to be accessed concurrently [3]. Therefore, this mode of sliding only relates to distant translocations on large DNA molecules by a limited subset of DNA-binding proteins [29,30].

## 3. Approaches for Observing Protein 1D Sliding

Bulk assays were initially designed and performed to monitor protein sliding. However, these methods usually provide indirect evidence and lack dynamic information; now, they have been largely overtaken by single-molecule experiments. Single-molecule imaging, especially single-molecule tracking using a total internal reflection fluorescence (TIRF) microscope, is capable of following a single protein and obtaining its trajectory on a DNA substrate. Other single-molecule methods, including force spectroscopy, also can be used for protein sliding investigation.

### 3.1. Biochemical Assays

Biochemical assays have been useful for measuring the speed and processivity of a polymerase or helicase. For example, by measuring the DNA products, Satoko Maki and Arthur Kornberg found that DNA polymerase III holoenzyme moved along the template strand at the rate of roughly one base every 2 milliseconds (ms) [31]. Using a helicase assay to detect DNA unwinding events, Frank G. Harmon and Stephen C. Kowalczykowski have determined the translocation rates of *E. coli* RecQ helicase on DNA [32]. The facilitated diffusion of restriction endonuclease also can be detected by electrophoretic mobility shift assay (EMSA). Rau and Sidorova have successfully determined the sliding rate of EcoRI and have demonstrated that the EcoRI likely performed a rotation-coupled diffusion along the DNA [10]. The method was based on measuring the ratio of the dissociation rate of protein from DNA containing one specific binding site to the dissociation rate from DNA containing two specific binding sites [10]. Remarkably, the formation of an MutS sliding clamp was initially discovered by biochemical assays in the 1990s. Gradia et al. have found that the MSH2–MSH6 complex (human MutS homologs) initially recognized a mismatch, while the ATP binding induced the complex to form a ring-like clamp on DNA [25,26]. Interestingly, this clamp has been later found to slide on DNA in a hydrolysis-independent way and eventually dissociated from DNA ends [25,26]. Although these assays have collected a great wealth of data, most evidence seem to be too indirect to describe the sliding processes and lack of kinetics as well as quantifications.

### 3.2. Single-Molecule Tracking

Over the past two decades, the developments of single-molecule techniques have enabled the observations of the individual behavior of a single particle, which eliminate the average effects during measurement and do not require synchronizations [33]. Single-molecule tracking that can follow a single fluorescent protein at a large time scale is ideal for measuring protein sliding along DNA.

The single-molecule tracking can be acquired using a TIRF microscope, such as a prism-type TIRF or objective-type TIRF [33]. A TIRF microscope allows the illumination of a thin region of the specimen (≈100–300 nm), which significantly increases the signal-to-noise ratio during imaging [24,33]. To image the fluorophore, flow chambers are often used by the assembly of a quartz slide, a piece of double-sided tape, and a coverslip, where the quartz slide has been treated with chemical reagents to produce a polyethylene glycol (PEG)/biotin-PEG passivated surface [34]. A motorized syringe pump is usually employed to deliver buffers or samples via controlled laminar flow [33].

**Figure 2:**
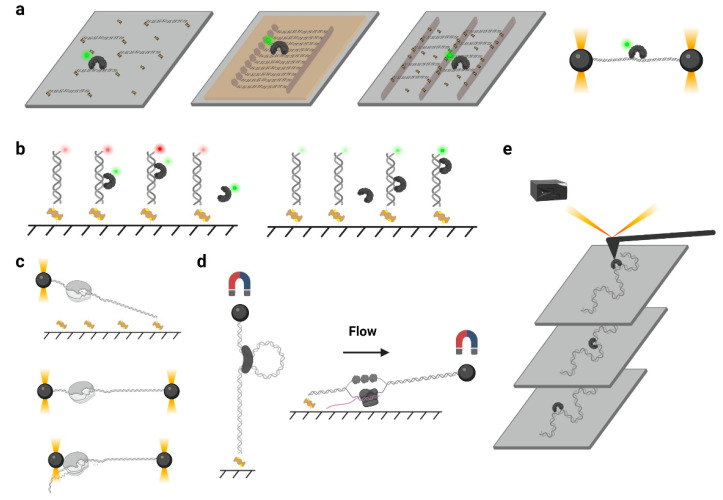
Single-molecule methods for observing protein 1D sliding. (**a**) An illustration of tracking a single protein molecule on DNA using TIRF or confocal microscopy. Four types of DNA immobilization methods are shown, including random immobilization, DNA curtains, DNA skybridges, and C-trap optical tweezers (from left to right). The gray rectangles represent the quartz surfaces and the yellow rectangle represents the lipid bilayers. The black spheres represent the dielectric beads. The fluorophore-labeled proteins (DNA-binding proteins with green dyes) on DNA are shown. (**b**) An illustration of monitoring the distance between DNA and protein using FRET (left) and PIFE (right). The fluorophore-labeled DNA substrates are immobilized on quartz slide surfaces. The DNA-binding proteins slide on DNA and cause FRET (left) or PIFE (right). (**c**) An illustration of detecting the forces associated with DNA–protein interactions using optical tweezers. Three types of experiment designs are shown, including surface-base assay, dumbbell-based assay using two optical traps to tether DNA double ends, and dumbbell-based assay using two optical traps to tether the DNA end and protein, respectively (from top to bottom). The black spheres represent the dielectric beads. (**d**) An illustration of detecting the forces associated with DNA–protein interactions using magnetic tweezers. A general set up (left) and a flow stretching set up (right) are shown. The black spheres represent the superparamagnetic beads. (**e**) An illustration of imaging the different states of DNA–protein interactions over time using AFM. The black box represents the detector which monitors the cantilever deflection.

In a typical single-molecule tracking experiment, the DNA is usually immobilized on the quartz surface via the biotin–neutravidin–biotin or digoxigenin–antibody interactions (Figure 2a). DNA molecules can be stretched by laminar flow and linked to the surface at both ends, or they can be linked at a single end and stretched by a continuous laminar flow. A similar method such as “DNA curtains”, which uses a combination of nanofabricated surface patterns and fluid lipid bilayers to align hundreds of DNA molecules (Figure 2a), has been developed recently [35]. However, some single-molecule experiments still suffer from the nonspecific protein binding on the surface, where the undesirable background signals significantly reduce the resolutions of the target proteins. Thus, a novel single-molecule tracking platform named “DNA skybridge” has been designed and applied to monitor proteins only on DNA (Figure 2a) [36]. Although the quartz barrier used in the DNA skybridge platform has not been commercially available, it is expected that most single-molecule tracking systems can be replaced by this surface-condition-independent and high-throughput method in future. In some single-molecule tracking assays, C-trap optical tweezers combining confocal fluorescence microscope have been used [37]. Instead of DNA immobilization on a quartz surface, the C-trap captures a DNA molecule by optical traps (Figure 2a), where the changes of DNA lengths and protein positions can be recorded simultaneously. While the C-trap instrument is now commercially available, one disadvantage is that only one molecule can be observed during a single experiment.

To monitor the DNA–protein interactions in real time, the fluorophore-labeled proteins can be injected into the chamber after DNA immobilization. For single-molecule tracking, the Cyanine (Cy3 and Cy5) and Alexa Fluor dyes, which are small probes that have both high quantum yields and high photostabilities, are recommended. To conjugate the protein with a fluorophore, methods include Cys-maleimide chemistry [33], hydrazinyl-iso-pictetspengler ligation [38], and sortase-mediated reaction [39], which only introduce small tags onto the proteins, and it has been shown that these can largely retain the biological activities of enzymes. In contrast, the commonly used fluorescent proteins are not recommended due to their large sizes and short lifetimes under TIRF illumination [40].

### 3.3. smFRET and PIFE

Single-molecule fluorescence resonance energy transfer (smFRET) has been widely applied in single-molecule studies. This approach uses a pair of donor and acceptor fluorophores to measure the distances between two biomolecules at 1–10 nanometer scales [33] (Figure 2b, left). Although this range of distance seems to be too narrow to observe a protein sliding on a large DNA molecule, appropriate strategy could be employed that the diffusion process still could be detected. For example, end blocked and Cy5-labeled DNA substrates were immobilized under TIRF to measure the DNA-binding by Cy3-MutS [41]. The diffused Cy3-MutS on the Cy5-DNA produced an FRET efficiency that could be clearly distinguished from the mismatch bound one. More importantly, the smFRET data have demonstrated that the ATP binding induced the mismatch-bound MutS switching into another mode of diffusion that had a much longer lifetime on DNA [41,42]. Later, the conformation of this long-life MutS has been determined as a sliding clamp on DNA [24]. smFRET also has been used to observe the sliding of a single-strand DNA-binding protein (SSB) on a single-stranded DNA (ssDNA) [43]. In this study, a ssDNA substrate containing 81 nucleotides (nt) was used, where the donor and acceptor fluorophores were separated by 69 nt. The diffusion of SSB along the ssDNA altered the distances between the donor and acceptor, which induced large FRET fluctuations in a millisecond timescale [44].

Protein-induced fluorescence enhancement (PIFE) also has been employed to study protein translocation. PIFE refers to a phenomenon when a protein binds very close to a fluorescent dye, where the local viscosity of the dye can be changed and leads to an increase in fluorescence intensity (Figure 2b, right). Compared with FRET, PIFE does not require protein labeling and has a detection range from 0 to 3 nanometers [45]. Myong et al. have constructed a 25 bp double-stranded RNA (dsRNA) with a DY547 labeled at one end [46]. They have observed a periodic fluorescence fluctuation when ATP and RIG-I protein were co-injected. The authors suggested that the RIG-I repetitively translocated along the dsRNA, which resulted in PIFE during single-molecule imaging [46]. Nevertheless, smFRET and PIFE have shown a relatively low resolution when they were used to determine the intermediate states of protein diffusion, and the trajectories of protein sliding along DNA cannot be directly measured. Thus, the combinations of multiple analysis are usually preferred.

### 3.4. Single-Molecule Force Spectroscopy

Single-molecule force spectroscopy approaches have emerged as useful tools to investigate the motions and forces associated with enzymatic activity. The optical tweezers, also known as optical traps, are one of the most versatile single-molecule manipulation techniques that can measure the force and the displacement of the trapped particle simultaneously with sub-nanometer resolution [47]. In a typical configuration of optical tweezers, a tightly focused laser beam exerts radiation pressure on a small dielectric bead, which is attached to the molecule of interest such as a DNA. In some experimental designs, the other end of the DNA is tethered to the surface (Figure 2c, top) or a second bead for stretching [48,49] (Figure 2c, middle). While the dsDNA maintains a longer extension distance than the ssDNA under low force (≈2 pN), the unwinding of dsDNA can be monitored in real time [50,51]. A DNA-binding protein, such as a polymerase, also can be attached to the second bead (Figure 2c, bottom), where the translocation of the enzyme on DNA can be observed [52]. Compared with other force spectroscopy methods, the optical tweezers have a much higher spatial resolution; however, they can only measure one molecule at a time. This drawback of low-throughput has limited its application in protein sliding investigation.

Another type of force spectroscopy is referred to as magnetic tweezers, which utilize a similar concept as optical tweezers. Instead of using the optical traps, magnetic tweezers contain magnets that provide an external magnetic field to manipulate dozens of magnetic particles simultaneously. In a general magnetic tweezers set up, a superparamagnetic bead is tethered to one end of a DNA molecule, while the other end of the DNA is attached to the slide surface [53] (Figure 2d, left). The external magnets are able to impart both twist and tension to the DNA, making magnetic tweezers an excellent method to study the motions of DNA translocases or topoisomerases [54,55,56]. Alternatively, a flow-stretching set-up has been designed, where the instrument combines the magnetic force and the drag force created by a laminar flow to hold the paramagnetic beads and stretch the DNA (Figure 2d, right). Using the flow-stretching assay, a number of DNA molecules have been tracked in real time, and the DNA replication, unwinding, and excision events were studied [57,58,59]. Due to the large volume of the magnetic field, the magnetic force spectroscopy is able to monitor hundreds of DNA molecules at one time but with lower spatial and temporal resolutions.

Atomic force microscopy (AFM) generates images by scanning a small cantilever with a sharp tip over the surface of a sample. By placing the tip in contact with the molecule of interest and moving the surface with respect to the tip, force can be modulated precisely, thereby changing the cantilever deflection. Cantilever deflection is further monitored by a laser beam reflected from the sharp tip onto a position-sensing detector [60] (Figure 2e). High-speed atomic force microscopy (HS-AFM) has been used to image the DNA–protein complex in real time, where the protein diffusion on DNA can be visualized [61]. Nevertheless, the main limitations of AFM come from the nonspecific interactions between proteins and the surface that may suppress the sliding [61].

## 4. Biological Significance of Protein 1D Sliding

Protein 1D sliding has been shown to be involved in many biological processes. Here, we summarize and discuss the significance of protein 1D sliding in biology.

### 4.1. Facilitation of Target Search

**Figure 3:**
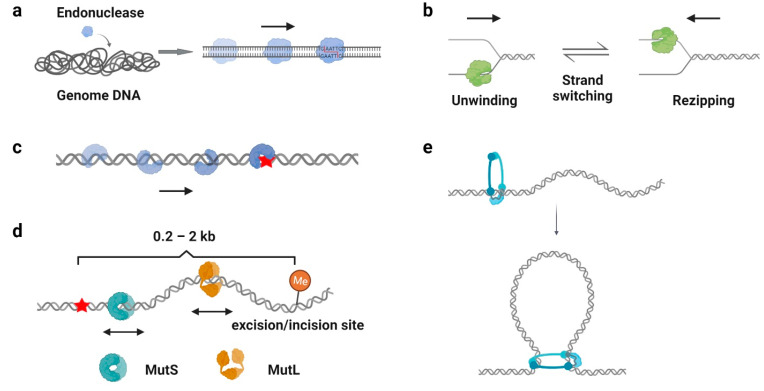
Biological functions of protein 1D sliding. (**a**) An illustration showing the process of an endonuclease targeting a sequence-specific site among the genome by facilitated diffusion. Arrows indicate the sliding of an endonuclease to a target site. (**b**) An illustration showing a helicase unwinding and rezipping on DNA. Black arrows indicate the directions of helicase translocation. (**c**) An illustration showing a DNA repair protein scanning along the DNA backbone and recognizing a damage site (red star). (**d**) An illustration showing the distant communications between a mismatch (red star) and an excision/incision site via MutS and MutL sliding clamps. Arrows indicate the sliding of MutS and MutL. (**e**) An illustration showing condensin binding to the DNA and extruding it as a loop to spatially organize the chromosomes.

One of the well-recognized functions of protein 1D sliding is to accelerate the target search process. For many DNA-binding proteins that specifically recognize particular sequences, such as transcription factors, endonucleases, and DNA methyltransferases, the target sites only constitute a minute fraction of the genome DNA (Figure 3a). Thus, the target search should be highly efficient. Actually, it is the case. Some of the proteins locate their target sites much more rapidly than the theoretical calculated one that uses a general diffusion–collision mechanism [19]. The search process was later modeled as a two-step event, where nonspecific protein–DNA binding first occurs and is followed by a 1D sliding to the target site. For example, the lacI repressor [19,20], tumor suppressor p53 [11,62], and endonucleases EcoRV, BcnI, and Endonuclease V [9,63,64,65,66] have been reported to slide along the DNA to facilitate the target search. Interestingly, while the continuous protein–DNA contacts without dissociation were initially believed to be essential for the searching processes [11,63,64], further evidence have shown that the sliding was accompanied by occasional hopping/jumping events, which helped the enzymes to locate their target sites [9,62,65,66,67,68,69,70]. The combination of sliding with continuous DNA contacts and hopping/jumping suggests a trade-off between speed and accuracy during the target search [68].

For some proteins that rely on guide RNA for targeting, 1D sliding is considered as lateral diffusion, which is a concept that originated from the lateral movements of lipids and proteins found in the membranes. Argonaute (AGO), a component of RNA-induced silencing complex (RISC), has been shown to perform both the facilitated diffusion and intersegmental transfer to accelerate RNA base pairing and bypass barriers [71,72,73]. Other RNA-guided endonucleases, such as the CRISPR-Cas9 and CRISPR-Cas12 systems, have been shown to slide on DNA as well [61,74,75]. However, a single-molecule study using DNA curtains has demonstrated that the CRISPR-Cas9 frequently located its targets by 3D diffusion [76], suggesting the contributions of 1D sliding of Cas proteins may be smaller than that of other DNA-binding proteins.

Protein complexes also have been shown to slide along the DNA for a target search. During homologous recombination (HR), the damaged DNA ends are first processed by strand resection to produce 3′ ssDNA overhangs [77]. Then, RecA/Rad51 forms helical filaments on the ssDNA, which are further aligned with a homologous dsDNA to initial repair. This progression is referred as the “homology search” [78]. Both the facilitated diffusion and intersegmental transfer of RecA filaments on dsDNA have been observed during the homology search [79,80]. Moreover, a recent single-molecule study further revealed that the Rad54 protein, a crucial HR factor that promotes strand invasion, could act as a motor driving ATP-dependent translocation to enhance recognition efficiency in homology search [81]. These data have demonstrated the indispensable roles of 1D sliding in DNA recombination.

### 4.2. Processivity Regulation

For most polymerases and helicases, it is obvious that the protein 1D translocation increases their processivity on DNA. However, sometimes, the 1D sliding can also stimulate a specific activity of the protein. For example, besides the helicase unwinding events that have been observed in bulk assays, single-molecule techniques have determined that many helicases undergo rezipping processes, which are enzyme-translocation-limited strand annealing events [51,56,82] (Figure 3b). The rezipping was later found to be induced by the conformational changes and strand switching of the helicase during DNA translocation [51,56], which can be regulated by SSB binding and other processivity factors [59]. This unwinding–rezipping control may help to limit possible long-range unwinding events that might lead to detrimental double-strand DNA breaks (DSBs) in cells [59]. In addition, *E. coli* RNA polymerase also has been found to employ a backtracking motion to proofread RNA synthesis [83].

As mentioned above (Section 2.3), β-clamp/PCNA forms a special ring-shaped conformation on DNA that provides a mobile platform to tether the DNA polymerase. While the replication sliding clamp itself usually adopts an intrinsically closed ring-like configuration, a sliding clamp loader belonging to the AAA+ ATPase family first binds ATP and recognizes the β-clamp/PCNA molecule to produce a cleft on the clamp [84]. Then, this complex binds a primer–template junction on DNA to stimulate the ATPase activity of the clamp loader [85,86]. ATP hydrolysis releases the loader from the β-clamp/PCNA and DNA, leading to the closure of the β-clamp/PCNA on substrate DNA [87,88]. Studies have demonstrated that the PCNA moves along DNA using two different sliding modes: it slides by tracking the DNA backbone in the rotational-coupled mode, while it moves at higher rates in the non-helical diffusion mode [89,90]. Most importantly, the replication sliding clamp exhibits a half-life of tens of minutes on DNA that enhances polymerase processivity by more than 1000-fold [91,92]. Other clamp-like proteins that have a long lifetime on DNA, such as the Mre11-Rad50-Nbs1 (MRN) complex, MutS, and MutL homologs, also have been shown to act as processivity factors during DNA repair [58,59,93,94].

### 4.3. DNA Duplex Interrogation

DNA damage occurs at a rate of 10,000 to 1,000,000 lesions per cell per day [95]. To maintain the stability of the genome, repair proteins have to recognize DNA lesions efficiently. Many DNA repair proteins have been reported to scan along the DNA for damage sites (Figure 3c), including the base excision repair protein glycosylases [96,97,98,99], mismatch repair protein MutS homologs [41,100], and nucleotide excision repair protein Rad4, XPA, and UvrB [101,102]. Among these studies, it is generally believed that the 1D sliding is used to follow the DNA backbone for lesion recognition, where a damage site only slightly destabilizes the helical structure of a duplex DNA [41,99,100]. In other words, the scanning processes likely involve rotation-coupled diffusions along the DNA helix, while the proteins are in continuous contact with the duplex DNA [41,103].

### 4.4. Distant Communications

In DNA repair pathways, 1D sliding has been employed to mediate the communications between multiple proteins. DNA mismatch repair (MMR) is an excision–resynthesis system that corrects mismatched nucleotides or insertion-deletion loops to maintain genomic stability [104]. While the MutS and MutL homologs are responsible for MMR initiation, the highly specific downstream MMR events rely on the distant communications between a mismatch and a strand discrimination site [105]. Although it has been previously established that the MutL homologs mediate multiple protein–protein interactions to connect mismatch recognition with strand incision/excision [106,107], the mechanism has not been revealed until recently. Single-molecule studies showed that MMR began with mispair search by a MutS dimer, where mismatch recognition triggered the formation of an ATP-bound MutS sliding clamp [25,41]. In *E. coli* that evolved DNA adenine methylation (Dam) and MutH as strand discrimination sources, MutS sliding clamps recruited MutL dimers onto the DNA by forming MutS–MutL complexes, where a single N-terminal domain (NTD) of MutL associated with MutS [24,108]. ATP binding induced the dimerization of MutL NTDs, leading to the formation of a MutL sliding clamp on the mismatched DNA [24]. Subsequently, the MutL sliding clamp recruited MutH endonuclease to introduce multiple strand breaks on the newly replicated strand as well as directed UvrD helicase to displace the error-containing DNA [24,59]. During *E. coli* MMR, both the MutS and MutL homologs utilize multiple sliding modes to achieve their functions: (1) Before mismatch recognition, MutS performs 1D diffusion along the DNA to search for a damage site [41]; (2) ATP-bound MutS adopts a similar configuration as β-clamp/PCNA to diffuse freely and maintain a long lifetime on DNA, which enables the loading of multiple MutS molecules from a single mismatch [109]; (3) The MutS–MutL complex moves with rotation-coupled diffusion along the DNA to have the MutL warped around the DNA helix [24]; (4) ATP binding induces the formation of a MutL ring-like clamp on DNA, where the MutL clamp slides with an extremely long lifetime on DNA to help MutH endonuclease search for a hemi-methylated site, as well as facilitate the UvrD helicase to excise the mis-incorporated strand [24,59] (Figure 3d). Another example of distant communications by 1D sliding is the PARP1, the member of the poly(ADP-ribose) polymerases (PARPs) family. The PARP1 undergoes auto-PARylation after damage recognition, which decreases the affinity of PARP1 for the lesion. This allows the protein to slide on DNA and thus facilitates the recruitment of other repair proteins [110].

### 4.5. Loop Extrusion

Large chromosomes are spatially organized as chromatin loops to promote and regulate important cellular functions, such as gene expression, replication, and segregation. Chromatin loops are formed by a process referred as “loop extrusion”, where the structural maintenance of chromosome (SMC) complexes (for example, condensin and cohesin) bind to the chromatin and extrude it as a loop [111] (Figure 3e). Recently, the DNA sliding of condensin and cohesin has been directly observed by single-molecule imaging [16,112,113]. Evidence supported that the ATP-hydrolysis-driven 1D translocations of condensin pulled the DNA into a foci, while flow stretching further demonstrated the formations of extruded loops at the foci [112,113]. Similar observations have been made for the cohesin, which established the cohesin–NIPBL as an ATP-driven molecular motor translocating along dsDNA [114,115,116].

## 5. Conclusions and Perspectives

Evolving experimental approaches, such as single-molecule techniques, as well as the computational modeling that have not been discussed here [117], have expanded our knowledge of how proteins modulate their motions on DNA. However, limitations remain in many single-molecule studies. For example, it seems to be difficult to image protein sliding with both high-resolution and high-throughput approach in a single experiment. Single-molecule tracking, which can monitor hundreds of individual molecules at one time, has a relatively low spatial resolution (about 200–300 nanometers). In contrast, optical tweezers usually retain a high spatial resolution (sub-nanometer) but only can study one molecule at a time. Another limitation among these studies is the use of DNA substrates that might be lack of physiological relevance. Most of the investigations employed stretched and naked DNA to image protein 1D sliding. These substrates are obviously different from the genomic DNA in cells that appear to be tangled and wrapped by nucleosomes. Future studies should focus on the preparation of DNA samples that are more physiologically relevant.

While the 1D sliding has been observed for many DNA-binding proteins, sometimes, it is still unclear if these motions are indispensable for their biological functions. Future investigations should focus on protein functional studies other than simply reporting the 1D sliding phenomenon. For instance, to figure out whether the facilitated diffusion is essential for the target search, mutations should be generated to exclusively abolish the diffusion process but retain the catalytic activity of the protein of interest. Alternatively, obstacles could be introduced on DNA to specifically hinder protein 1D diffusion. Next, quantitative functional assays can be performed to evaluate the contributions of facilitated diffusion during target search.

Understanding the mechanisms of protein 1D sliding can also help to identify and predict mutations that cause human diseases. A recent study of human MLH1-PMS2 has demonstrated that the heterodimers employ the N-terminal ATP-binding domains, the C-terminal dimerization domains, and flexible linkers to form a closed ring-like clamp diffusing along the DNA [27]. Mutations on the N- and C-terminal domains that disrupt the sliding clamp formation result in the loss function of MLH1-PMS2, which leads to Lynch Syndrome in humans [27]. In contrast, the MLH1–PMS2 disordered linkers primarily supply the flexibility to wrap around the DNA; thus, no pathogenic missense mutation within the linker domain has been found [27].

Although a lot of challenges remain, future studies that are capable of deciphering how protein 1D sliding promotes the efficiency and specificity of a biochemical reaction likely offer the potential for resolving complicated biological problems, such as the MMR distant communications that are mediated by multiple sliding clamps.

## Data Availability

Not applicable.
